# Relationship between mRNA secondary structure and sequence variability in Chloroplast genes: possible life history implications

**DOI:** 10.1186/1471-2164-9-48

**Published:** 2008-01-28

**Authors:** Neeraja M Krishnan, Hervé Seligmann, Basuthkar J Rao

**Affiliations:** 1 Department of Biological Sciences, Tata Institute of Fundamental Research, 1 Homi Bhabha road, Colaba, Mumbai 400005, India; 2 Department of Evolution, Systematics and Ecology, The Hebrew University of Jerusalem, Jerusalem 91904, Israel; 3 Department of Life Sciences, Ben Gurion University of the Negev, 84105 Beer Sheva, Israel

## Abstract

**Background:**

Synonymous sites are freer to vary because of redundancy in genetic code. Messenger RNA secondary structure restricts this freedom, as revealed by previous findings in mitochondrial genes that mutations at third codon position nucleotides in helices are more selected against than those in loops. This motivated us to explore the constraints imposed by mRNA secondary structure on evolutionary variability at all codon positions in general, in chloroplast systems.

**Results:**

We found that the evolutionary variability and intrinsic secondary structure stability of these sequences share an inverse relationship. Simulations of most likely single nucleotide evolution in *Psilotum nudum *and *Nephroselmis olivacea *mRNAs, indicate that helix-forming propensities of mutated mRNAs are greater than those of the natural mRNAs for short sequences and vice-versa for long sequences. Moreover, helix-forming propensity estimated by the percentage of total mRNA in helices increases gradually with mRNA length, saturating beyond 1000 nucleotides. Protection levels of functionally important sites vary across plants and proteins: *r*-strategists minimize mutation costs in large genes; *K*-strategists do the opposite.

**Conclusion:**

Mrna length presumably predisposes shorter mRNAs to evolve under different constraints than longer mRNAs. The positive correlation between secondary structure protection and functional importance of sites suggests that some sites might be conserved due to packing-protection constraints at the nucleic acid level in addition to protein level constraints. Consequently, nucleic acid secondary structure *a priori *biases mutations. The converse (exposure of conserved sites) apparently occurs in a smaller number of cases, indicating a different evolutionary adaptive strategy in these plants. The differences between the protection levels of functionally important sites for *r*- and *K-*strategists reflect their respective molecular adaptive strategies. These converge with increasing domestication levels of *K*-strategists, perhaps because domestication increases reproductive output.

## Background

The structure of the genetic code imposes constraints on the evolutionary variability of a nucleotide in a gene sequence, depending on its codon position. The third codon positions are almost freely variable, because of the redundancy in the genetic code. The excess variability at the third codon positions, after accounting for the replication-caused effects could be explained by mRNA secondary structure, in mitochondrial genes [1,2].

Therefore, a third codon position nucleotide in a loop is more variable than that in a helix. This suggests additional evolutionary constraints imposed by the mRNA secondary structure, which motivated us to study its relationship with evolutionary variability. Note that the specific structure of the genetic code maximizes the capacity of coding regions to form helices in their secondary structure, while simultaneously also maximizing occurrences of off-frame stops [3]. While maximizing numbers of off frame stops minimizes costs of ribosomal slippages [4,5], the rationale for maximizing propensity for secondary structure is not known. We suggest that it might protect from mutations, and test this hypothesis.

We chose a dataset of chloroplast mRNAs for our secondary structure analyses. This is because regulation of plastid gene expression during development and in response to light is of substantial interest in plant biology. Understanding the key factors underlying these processes can yield important insights that can be used to engineer and transform plastids [6]. We use mFold, a free-energy minimization algorithm that uses sequence-dependent thermodynamic parameters, for evaluating the sub-optimal range of secondary structures for chloroplast mRNAs.

We found that the secondary structure stability correlated negatively with the percentage of variable sites in the gene. In order to gain further insight on the mRNA secondary structure and sequence relationship, we decided to simulate sequences by mutating mRNAs at each site according to their most likely substitution rate category. This categorization for each site was calculated using the 'dnaml' tool from PHYLIP v. 3.8.0. package. Our simulation analyses revealed mRNA size as an important factor, which acts as a flip-flop switch control on how these mutations affected the secondary structure.

Transcript length has already been reported to be negatively correlated with gene expression level (mRNA abundance), positively with protein divergence in Drosophila [7] and metabolic cost minimization of protein synthesis [8], and other factors relevant to molecular evolutionary ecology [9]. We found that for shorter sequences, a greater percentage of the secondary structure of mutated mRNAs is composed of helices, than that for the natural mRNAs. Whereas for longer sequences, the secondary structure of mutated mRNAs is composed of lesser percentage of sites in helices, than that for natural mRNAs.

It is possible that secondary structure (i.e. being part of helices) usually protects functionally important sites from mutations. This possibility is also ascertained by the fact that spontaneous mutations occur more rapidly on single-stranded DNA than double-stranded DNA [10]. This could bias the mutational spectrum of a gene towards micro-adaptive changes, as mutations at less important sites are more likely to produce functional proteins with slightly altered optima. The opposite is also a potential adaptation, to increase adaptability to more drastic environmental changes and in life-history. The former fits better species considered as overall *K*-strategists because it increases micro-adaptations and niche-specialization, while the latter fits overall *r-*strategists, as its costs of decreasing survival are more likely to be bearable by organisms with high numbers of off-spring. Our results fit these general principles, and justify further adequate analyses. This approach towards molecular processes and evolution converges with that described for *r*- and *K*-strategists at whole organism level by Skulachev [11].

## Results

### Secondary structural folding stability of nucleic acid sequences modulates their evolutionary variability

**Figure 1:**
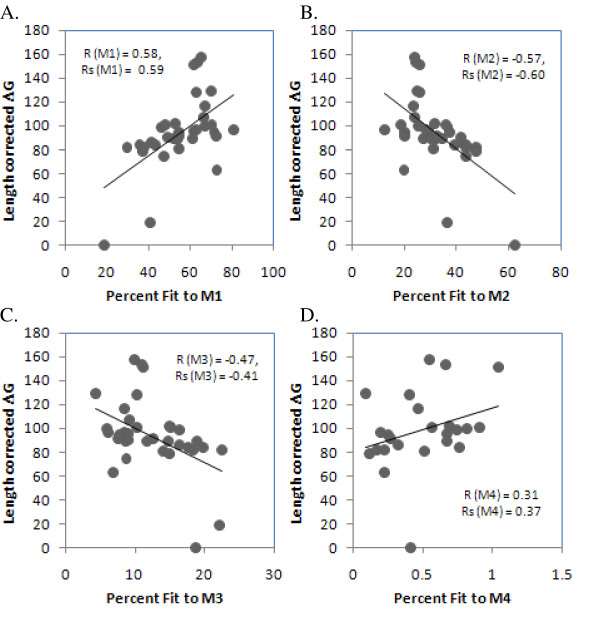
**Correlation of residual length-adjusted RNA secondary structure stability (-ΔG) with that gene's most likely fits to substitution rate categories**. The X-axes represent the percent of sites in 35 genes fitting four predefined rate categories with increasing average rates: 1A. M1 (average rate parameter = 0.1), 1B. M2 (average rate parameter = 0.2), 1C. M3 (average rate parameter = 0.3) and 1D. M4 (average rate parameter = 0.4), and the Y-axis represents the residual length-adjusted secondary structure stability (-ΔG) of these genes folded as RNAs. We averaged the folding stabilities over alternative structures that were within 50% optimality of the most stable structure; these average stabilities were further averaged across all seventeen species. Residuals were calculated by treating length as the independent variable and the negative of stability (-ΔG) as the dependent variable (see Methods).

We tested for correlations between the length-adjusted residual stability measure for each gene (see Methods) and that gene's relative fit to four pre-defined substitution rate categories (M1, M2, M3 and M4). This estimate of stability correlated positively (Pearson correlation coefficient r = 0.55, P = 0.0006; rs = 0.54, P = 0.0007; Figure 1A) with the corresponding gene's percent fit to the slowest substitution rate category M1 and negatively with the percent fits to faster substitution rate categories M2 (r = -0.60, P = 0.0006; rs = -0.59, P = 0.0002; Figure 1B) and M3 (r = -0.39, P = 0.0408; rs = -0.34, P = 0.05; Figure 1C). Correlations of residual RNA stability with percent fit to the highest rate category M4 were also positive but not statistically significant (r = 0.30, P = 0.17; rs = 0.37, P = 0.08; Figure 1D) because only 24 out of 35 genes contain sites fitting M4 category. Overall, these results indicate that the chloroplast sense strand RNA sequences with highly variable sites fold into less stable secondary structures, and corroborate similar findings in primate mitochondrial protein coding genes [1,2,12]. The slight differences between the correlation coefficients for parametric and non-parametric analyses are mainly due to minimization of extreme data point effects by non-parametric analyses. All P-values are according to 2-tailed t-tests.

**Figure 2:**
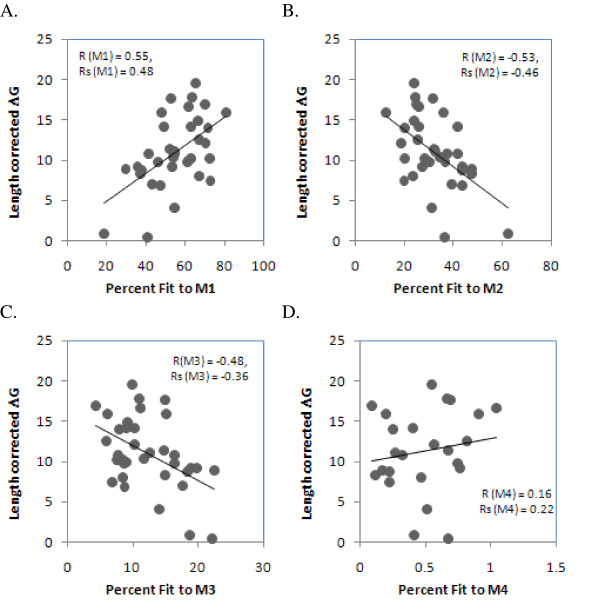
**Correlation of residual length-adjusted DNA secondary structure stability (-ΔG) with that gene’s most likely fits to evolutionary variability categories.** The X-axes represent the percent of sites in 35 genes fitting four variability categories as described in Figure 1: M1, M2, M3 and M4 (2A, 2B, 2C and 2D) and the Y-axis represents the residual length-corrected DNA secondary structure stability, obtained using the DNA folding version of mFold software. Averages of stabilities and residuals were calculated as in Figure 1.

The DNA stability of the sense-strand sequence bore similar significant results with percent fits of the genes to two among four rate categories (Figure 2). The correlation coefficients of DNA stability with percent fits to M1, M2, M3 and M4 were 0.50, P = 0.0024; -0.53, P = 0.0013; -0.34, P = 0.0513 and 0.161, P = 0.474 respectively. Non-parametric Spearman's rank correlation analyses yielded significant results for percent fits to M1 (rs = 0.484, P = 0.003), M2 (rs = -0.464, P = 0.005) and M3 (rs = -0.361, P = 0.033) but not to M4 (rs = 0.224, P = 0.291). It is not clear whether these subtle differences in the strength and significance of correlations for the RNA and DNA strands are due to an inherent difference in the robustness of the respective folding programs or indeed reflects different genuine evolutionary interactions between RNA and DNA secondary structures and sequences. However, the similarity between the trends for RNA and DNA strands confirms that this correlation is due to effects specific to underlying single-strandedness. DNA-specific effects might associate with protection from mutations during single stranded periods, such as during DNA replication [1,2] or mRNA transcription [13-17]. The notably stronger RNA-associated effects could relate to protection from decay [18], as well as alterations of the sequence that would change the coding properties of the mRNA.

### Variability Distribution at Synonymous and Non-synonymous sites

**Figure 3:**
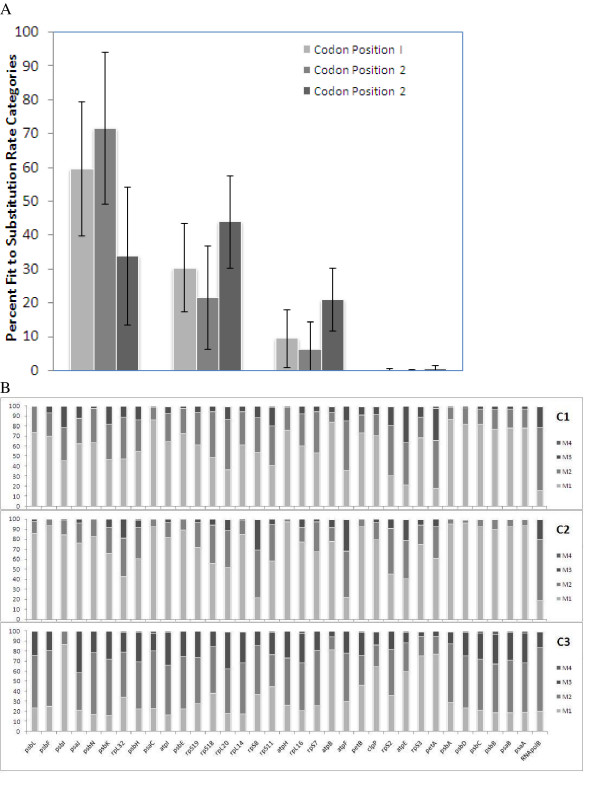
**Variability distributions at synonymous and non-synonymous sites**. The relative fits (%) to the four variability categories (M1, M2, M3 and M4) are 3A. averaged across and 3B. shown individually at the three codon positions for all 35 genes in an ascending order of their sizes. The standard deviations of the relative variability categories are indicated as error bars in 3A.

In order to clearly interpret the above results in the light of synonymous/non-synonymous site variation, we analyzed the distribution of sites at all three codon positions among the four chosen substitution rate categories (Figure 3A). The percentage fits to these four categories were averaged across genes, individually for each codon position. We have plotted these averages along with the standard deviation bars of these estimates. The non-synonymous sites, i.e. sites at first or second codon position, are more prevalent as M1-fitting sites. Second codon positions fit the M1 category better than first codon positions, as expected by its greater functional relevance [19]. The third codon positions are relatively more prevalent as M2-, M3- and M4-fitting sites.

**Figure 4:**
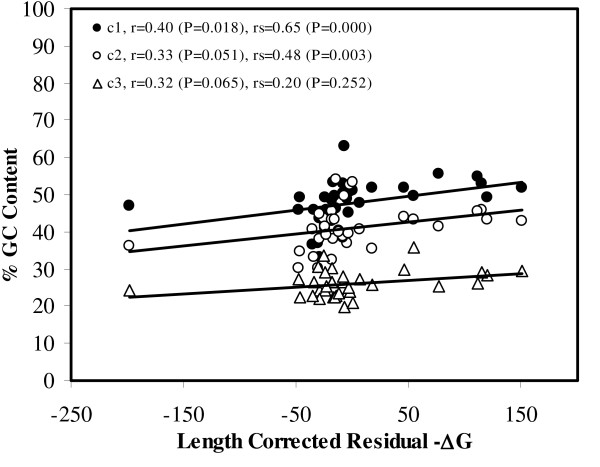
**GC content at synonymous and non-synonymous sites correlates positively with residual, length-adjusted stability**. The Y-axis is the relative G+C content (%) averaged across all 35 genes, separately at the three codon positions. The X-axis is the residual length-adjusted stability (-ΔG), calculated as in Figure 1. The parametric correlation coefficients (r) and non-parametric Spearman's rank correlation coefficients (rs) are indicated.

Chloroplast mRNAs are GC-rich, more at first and second codon positions, than at third codon positions, which contain more A's and T's (Figure 4). The average GC content at the first and second codon position of mRNAs correlates positively with their residual length-adjusted stability (Spearman's rank correlation coefficient rs = 0.65 and 0.48 (P = 0.000 and 0.003, 2-tailed t-tests)) as shown in Figure 4. The GC content at the third codon position does not correlate with the residual stability. Overall, this is compatible with previous finding that a negative correlation exists between substitution rates at the first two codon positions versus the third codon position, for GC content [20]. These results confirm that variability that negatively affects mRNA stability primarily arises at synonymous sites because these sites tend to fit M2, M3 and M4 more than the non-synonymous sites. The argument becomes circular: the synonymous sites being GC-poor, *a priori *contributed lesser to mRNA stability and thereby, have lesser evolutionary conservation constraints.

There is considerable variation among genes in the distribution of variability levels at the synonymous and non-synonymous sites, as indicated by the standard deviation bars. Our conclusion qualitatively remains the same even after separately analyzing this average distribution for individual genes (Figure 3B). However, the variability distributions for individual genes reveal slightly distinct patterns between the photosystem subunits and the ribosomal protein and ATPase subunits. In photosystem genes, 71% and 86% of first and second codon position sites fit M1, whereas in ribosomal protein and ATPase subunits, these are 55% and 63% respectively. As a result, fits to the higher variability categories M2 and M3 were approximately 1.5–2 and 1.9–6 fold respectively, at the first and second codon positions, for the ATPase and ribosomal protein subunits than for the photosystem genes (M2; M3 fits for the ATPase and ribosomal proteins: 34; 11% and 27; 10% and for the photosystem genes: 23; 6% and 12; 2%, at the first two codon positions respectively).

### Local changes in secondary structure upon mutations in Psilotum nudum and Nephroselmis olivacea: Effects of mRNA size

**Figure 5:**
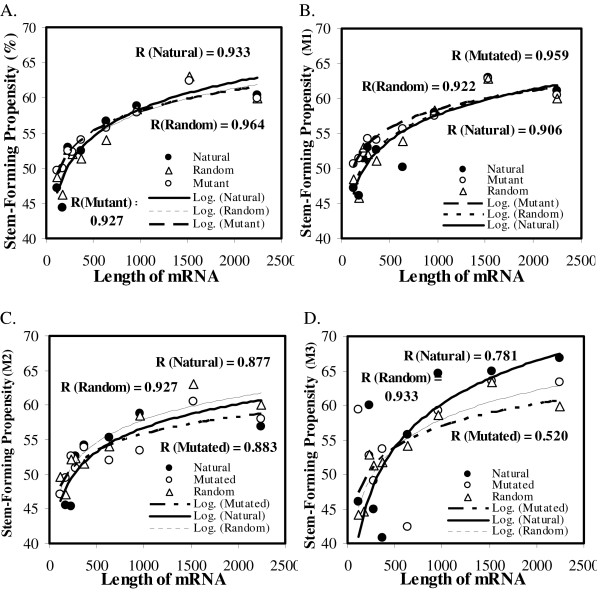
**Stem-forming propensities of mRNAs vs. length of the mRNA for natural, random and mutated sequences for A. overall mRNA, B. M1-fitting sites, C. M2-fitting sites, and D. M3-fitting sites**. Nine mRNAs (psbF (120 bases), psbK (177 bases), rps18 (227 bases), rps9 (278 bases), rpl14 (368 bases), rps3 (641 bases), pet A (965 bases), psbB (1526 bases) and psaA (2244 bases)) belonging to the species *Psilotum nudum *(NC_003386) were considered. For mutated and random mRNAs, each point represents average stem-forming propensity over fifty such mutated and randomized sequences respectively and over sub-optimal alternative structures with at least 50% stability of the optimal structure for each mutated sequence.

We compared helix-forming propensities from nine mRNAs of different lengths in *Psilotum nudum *and *Nephroselmis olivacea *among natural, mutated and randomized sequences (see Methods for details). These comparisons were performed after considering all the sites together as well as separately, based on the rate category of sites: M1-, M2-, M3-, and M4-fitting (see Figures 5A–D and Additional File 1). Only three genes (psbA, petA and rpS3) contained sites that fit the M4 category, and therefore, it was not possible to show the trend in this case.

In general, for all types of sequences (natural, mutated and random), the helix-forming propensities of mRNAs increase with length of the mRNA up to a given length and then saturate for longer mRNAs, as indicated by the gradual logarithmic trend (see Figures 5A–D and Additional File 1). The overall integral stabilities (-ΔGs) of mRNAs follow a linear trend versus the size of the mRNA (see Additional File 2) but the average percentage of sites in these mRNAs that form helices correlate logarithmically with integral mRNA stability (see Additional File 2). This explains the saturation effect we observe in Figure 5 (and Additional File 1; see Discussion). Upon mutating mRNA sequences under the four site-specific substitution rate categories (M1, M2, M3, and M4) and imposing transition: transversion bias of 2:1, we found that the difference between the overall helix-forming propensities of mutated and natural mRNAs varies according to their size. The mutated mRNA helix-forming propensity is greater than that of the natural mRNAs for shorter transcript sizes, and the trend reverses for longer mRNAs thus generating a crossover point in the correlation trend (see Figures 5A–D and Additional File 1). The difference between the natural and mutated helix-forming propensity curves is significant for all sites considered together (t = 3.155, P = 0.016; 2-tailed t-test) as well as for M1-fitting sites (t = 2.719, P = 0.030; 2-tailed t-test), but not for the sites fitting the remaining rate categories. The differences between natural and random or between random and mutated helix-forming propensities are not significant for any of the cases, not even for the entire mRNA. This is particularly interesting, considering that mutating sequences results on average, 17% nucleotide changes, whereas randomizations result in 73% nucleotide changes (very close to the expected 75% changes when considering each nucleotide as equi-probable) as compared to the original mRNA sequence. These length-dependent randomization effects on RNA secondary structures might explain contradictions in the field on effects of sequence randomization on RNA folding stabilities. Some report greater stability for natural than randomized mRNA sequences [21], while some report contrarily [22].

### Protection from mutations at gene levels

The correlations between evolutionary variability of sites and stemminess of those sites for the 35 homologous genes in all seventeen chloroplast genomes are described in a table (see Additional File 3). In a statistically significant majority of genes (23 among 35, P < 0.05 according to 1-tailed sign test), conserved sites form more secondary structure than variable sites. This tendency was significant for 11 specific genes (atpH, clpP, psaA, psaB, psbA, psbB, psbC, psbD, rps19, rps2, rps8), which is more than 6 times the 1.75 statistically significant results expected according to the multiplicity of tests at P < 0.05. In four genes (petA, rpl14, RNApolB, rps18), the opposite was true: in most plant species, evolutionary conserved sites had lesser helix-forming propensity than other sites. This number is also twice more than the expected number of false positive results at P < 0.05. Hence, results suggest that in a majority of cases, sites coding for functionally important (low variability) protein regions are protected from mutations by mRNA secondary structure (high stemminess, see *Methods *section for definition), but that in specific cases, also the opposite occurs. Statistically, positive and negative associations between site conservation and formation of stems can be considered as existing and are not artifacts.

We did not detect significant differences between these associations for genes coded on the sense strand versus those coded on the anti-sense strand, not even for any particular species, despite the differences between the nucleotide contents for these strands.

The correlations between site variability and helix-formation was significant at P < 0.05 (2 tailed tests, both directions) in 51 among 595 (8.57%, 36 cases negative (protecting conserved sites), 15 cases positive (exposing them)) mRNA-species combinations, which is twice the 2.5% expected by pure chance for negative, but barely more (2.752%) for positive correlations. If hypotheses were available for expecting the direction of these correlations and one tailed tests were used, 27 and 58 cases would be significantly positive and negative, respectively. This number is more than expected by pure chance for negative correlations, but not for positive ones. However, it is clear that some mRNAs have more significant correlations than expected, and others avoid them, including for positive ones. Indeed, a binomial distribution would expect in these conditions, 16.6% of the mRNAs to have no significant correlation in any of the 17 species, but in fact 34.28% (12 out of 35) of the mRNAs had no significant correlations in any species. On the other hand, there were several specific mRNAs, for which more species bore significant (p < 0.05) correlations than expected by a binomial distribution. This result suggests that in some mRNAs, evolutionary pressures tend to create such correlations, and in others, pressures prevent them. Considering that results suggest the existence of two forces, one occasionally causing positive, and another occasionally negative associations at the level of whole mRNA sequences, one should not be surprised that in a larger than expected number of cases, balance between forces results in no or low, non-significant correlations between site-specific mRNA secondary structure and variability levels. These principles were also qualitatively correct while considering numbers of significant cases according to one tailed tests, separately for positive and negative associations. Hence, some specific mRNAs tend towards negative (protecting conserved sites against mutations), a smaller group towards positive (exposing conserved sites to mutations), and a third group towards no association between secondary structure and site conservation (which could also mean a mixture of the two previous strategies for different regions of the same mRNA). Investigating the rationale behind this trimodal distribution of mRNAs is beyond the scope of this project, but suggests different adaptive strategies for different protein groups, and for different types of sites or amino acids. Results also suggest that the pressures causing associations between mRNA secondary structure formation and site conservation are ubiquitous, and exist also in mRNAs where no association is detected due to balancing forces.

### Protection from mutations at species levels

At the level of species, evolutionary variability of sites associated negatively with stemminess in a statistically significant majority of mRNAs for 14 among 17 species (sign test, P < 0.05). At the level of single species, this tendency was significant at P < 0.05 (1-tailed test) for *Chlamydomonas reinhardtii, Chlorella vulgaris, Mesostigma viride, Physcomitella patens patens, Triticum aestivum *and *Psilotum nudum*, which is more than the number of species expected by pure chance. There was no species for which a significant number of mRNAs had the opposite association. *Calycanthus floridus *was the most extreme in this direction, with 40% of its mRNAs showing the positive association between evolutionary variability at a site and stem formation by that site. There was no species for which no mRNA had a significant correlation between site variability and helix-formation, which is less than what would be expected. There were more species with no significant positive correlations between site variability and helix-formation than expected, which suggests that in some species, strong positive correlations are avoided (exposing conserved sites to mutations is avoided). There were lesser species with no significant negative correlations between site variability and stem formation than expected, which suggests that some species tend to have many strong negative correlations (protection of conserved sites from mutations). These results suggest that protein types and plant species vary with respect to correlations between site conservation and helix-formation. In some cases, exposure to, rather than protection from mutations, is evolutionary adaptive, at least in some mRNAs, such as RNApolB, where there was a positive correlation in 15 among 17 species, statistically significant at P = 0.05 (2 tailed t-test) for 7 species.

We found that overall, protection from mutations decreases with gene length by calculating the correlation between gene length and the correlation coefficient between site variability and helix-formation for that gene. This correlation was positive for 31 among 35 mRNAs (p < 0.05, sign test), and significant according to a two tailed test for 6 mRNAs, among which none is negative. Hence, overall, for homologous genes, the extent of protection of conserved sites decreases with gene length. A superficial look at these results could suggest that the lower correlations for longer mRNAs are due to lower accuracy of mFold predictions for longer sequences. According to this rationale, the longer mRNA homologues would have the least negative t values for correlations between site variability and helix-formation, and these would be close to zero (meaning low statistical significance, presumably due to prediction inaccuracies).

However, this rationale does not hold; in several mRNA species where the decrease with gene length was significant, the longer mRNA homologues do not have t statistics that are closer to zero or have low statistical significances, but they are often positive and sometimes, even statistically significant (see for example rpl20 and petL). This result is not compatible with random errors due to folding inaccuracies, and hence the hypothesis that prediction inaccuracies increasing with sequence length can explain these results seems less likely than the one indicating that these results reflect biological phenomena. It is possible that the decreased correlations for longer homologues reflect that their greater lengths are not due to additions of functionally necessary regions, such as catalytic sites, but perhaps, due to addition of sites with species-specific regulatory functions, or with no or little functional importance.

At the level of single species, across genes, the level of protection of conserved sites decreased with gene length in 11 species, significantly so, for *Spinacea oleracea *and *Adiantum capillus-veneris*. This was more the case for larger "vascular" plants that can be considered broadly as *K*-strategists as compared to "micro" plants (algae, bryophytes, etc..., t test, P < 0.05). Within vascular plants, these tendencies decreased with levels of domestication for 20 among 35 genes, significantly so at P < 0.05 for 7 genes and increased significantly only in one mRNA (Spearman rank correlations, 2 tailed tests).

This suggests that artificial selection made genes of these relatively *K*-strategy oriented plants resemble more those of *r*-strategists. Overall, these tendencies are clearer while considering data across species for specific mRNAs than while analyzing across mRNAs for specific species, but the same principles are valid at both levels, although weaker for species-specific analyses. Hence, results suggest more mRNA-specific adaptations than species-specific ones.

## Discussion

### Accuracy and inaccuracy of Folding Prediction Programs

The assumption that secondary structure predictions are essentially accurate is central to this study, and hence it is worth dwelling on some evidence justifying it. We will also discuss subsequently how the known inaccuracies of folding prediction programs are unlikely to bias our results.

Real functional RNAs are known to and also predicted to have fewer alternative structures than randomized sequences [23,24]. This already establishes a level of realism in the details of the predictions. A prediction accuracy of 90% reported for intermolecular hybridization of RNAs, indicates at least a similar accuracy for our simpler case of intra-molecular RNA hybridization [25,26]. Combinations of free energy minimization and comparative sequence analysis as used here, are more effective in enhancing structure prediction accuracy by finding a common low free energy structure [27-29].

Inaccuracy in secondary structure prediction algorithms is more prominent while having to predict a unique functional structure and is in fact minimized while considering a distribution of sub-optimal secondary structures. Nevertheless, at least in some cases where secondary structure was experimentally authenticated, specific local stem-loop structures are accurately predicted by folding programs for functionally important secondary structure components in chloroplast mRNAs [30]. In addition, structural predictions become more inaccurate with increase in the length of the sequences because of the concomitant increase in possible folds for longer RNAs and in particular, when the contact distance between the base pairs exceeds 100 nucleotides [31]. In theory, a Boltzmann's ensemble of secondary structures, predicted by programs such as Sfold [32,33] and Vienna [34,35] would better reflect the population of structures for any given mRNA. However, the temperature of plants is widely determined by their environment. The properties of the distribution of secondary structures are determined by temperature. Hence, precise calculations using Boltzmann's distribution should integrate temperatures over the range and frequency encountered by each plant species in its natural environment. Therefore, while calculations using Sfold's Boltzmann distribution predictions are more precise and preferable for homeotherms, less accurate calculations apparently perform equally well for poikilotherms: predictions of tRNA structures by mFold and Sfold in poikilothermic lizards yield similar results [36].

There are other factors that are not accounted by classical secondary structure prediction algorithms. One of them is the formation of protein-mRNA complexes (including in chloroplasts, [37]). The other is the formation of non-canonical base pairs participating in the functional secondary structure of crucially important RNAs [38]. These considerations are important and not considering them probably adds noise to the analyses we present. In other words, if it was possible to integrate in our analyses accurate information on secondary structure stabilization via complex formation with proteins, and/or on non-canonical base pairs, we would detect more statistically significant results than what we can detect now. It is much less likely that including that information would prevent observing the patterns we describe. However, if it was so, this would mean that these additional interactions (such as complex formation with proteins) are designed to prevent the patterns we describe. In that case, these would be two counter-balancing mechanisms, each important for RNA function and worth describing. It is also clear that even RNAs that form complexes with proteins are only part of the time in such complexes, and hence our results might be relevant in this more restricted context for some of the RNAs. The relevance of non-canonical base pairings to our specific issue is lesser: such base pairings have been described until now mainly in rRNAs and other RNA molecules where RNA and its secondary structure play a prime functional role. It is unlikely that this is a major factor for typical mRNAs, because their function is probably less confined to specific secondary structures, but rather more to group of alternative, closely related secondary structures with relatively low folding stabilities. However, it makes sense to consider that in yet not described specific cases, non-canonical base pairings may play an important role in determining the secondary structure and therefore, function of the mRNA. The roles for mRNA secondary structure that we detect, while considering the simpler scenario without non-canonical base pairings, only ascertain that further accurate secondary structure predictions would be very valuable to better understand RNA biology, especially while integrating cellular and whole organism level physiologies with evolutionary perspectives.

### Secondary structural stability modulates Sequence Variability

We do not detect significant correlations between integral mRNA stability and transcript's evolutionary variability. This might be because evolutionary variability, quantified by a percentage is already size-adjusted, while integral stability is not.

However, integral and length adjusted residual stabilities increase with the absolute number of sites fitting each substitution rate categories (see Additional File 4; integral stability: Spearman's rank correlation coefficients rs = 0.952, 0.821, 0.797 and 0.905; residual stability: rs = 0.684, 0.578, 0.531 (P = 0.001) and 0.535 (P = 0.001) for M1, M2, M3 and M4 respectively). This result, for the least, stresses that the evolution of RNA stability is in part explained by length, and mostly by the content and context-dependent component of the RNA. It is very unlikely that qualitatively our conclusions are due to artifacts (increase in inaccuracies of mFold predictions with RNA length), because correlations between evolutionary variability and both stabilities (integral and size adjusted) yield similar results.

Evolutionary variability (percentage of sites fitting M2 and M3 categories) of nucleotide sequences decreases with residual, length-adjusted stability of the gene, when folded as RNA and DNA. This would indicate that the helix-forming regions accumulate lesser mutations than loops [1,2,12], which is in agreement with the positive correlation observed between presence of structural motifs and thermodynamic secondary structure stability [39]. The M1-fitting sites are significantly over-represented at the first and second codon positions than at the third codon positions, whereas the M2-, M3-, and M4-fitting sites are relatively over-represented at the third codon positions (Figure 3A). The relatively high GC content at the first and second codon positions (Figure 4), explains well the positive correlation between the % M1-fitting sites and secondary structural stability. Similarly, the negative correlations of secondary structure stability with M2- and M3-fitting sites can be explained by the lower G+C content at the third codon positions. The relative AT richness at the third codon positions has been reported to be caused by codon usage bias in newly reported chloroplast genomes of *Nuphar advena *and *Ranunculus macranthus *species [40] as well as in angiosperms like *Oryza sativa*, *Zea mays*, *Triticum aestivum *and *Arabidopsis thaliana *[41]. Codon usage bias is known to impose a variety of functional constraints by favoring increase mRNA stability [42], altered splicing [43,44], hidden stop-codon formation [45] and translational efficiency/gene expression often in relation to codon-anticodon matching frequencies from existing tRNA pool [46-51]. It is also known to be controlled by genome-wide mutational constraints, probably due to underlying molecular processes [52,53]. This suggests that codon usage bias may also be acting as a concerted component in shaping the positive association between RNA secondary structural stability and evolutionary variability.

Although our analyses yield similar negative correlations between sequence variability and folding stabilities for both RNA as well as DNA, we will be primarily discussing our results in the context of RNA. We do this because the steady state level of RNA in a cell is likely to be much higher than that of single-stranded DNA. Further, the DNA folding program of mFold server [54,55] is currently not as well developed as the RNA version in terms of its incorporation of thermodynamic parameters and hence, stabilities obtained from the two versions are not comparable. In a study testing the mutational robustness of over 1000 naturally and artificially selected RNA structures, mutation-proneness correlated negatively with thermodynamic stability in the selected RNA molecules [56]. Our observed negative correlation of sequence variability with secondary structure stability is highly consistent with their results. These non-intuitive results suggest the acquisition of DNA mutations by the RNA during transcription, thereby affecting its intrinsic stability, half-life, and ultimately, protein turnover. These properties strongly suggest that RNA stability is intimately connected with the functional regulation of the molecule in the cell.

### Effects of mRNA length on mutational stability of secondary structure

We examined the effect of mRNA length on its mutational stability in a randomly chosen species, *Psilotum nudum*, and for assessing generality of results, a species distantly related to it, *Nephroselmis olivacea*. In both species, mRNA length associates with mutational stability (see Figure 5 and Additional File 1). Structural RNAs such as ribosomal and nucleolar spliceosomal RNAs and transfer RNAs have a lower stability than a random RNA sequence with the same dinucleotide frequency [57]. In our analyses of the mutational stability of chloroplast mRNAs and the non-randomness in intrinsic mRNA stability, it is possible that the smaller mRNAs behave like tRNAs or structural RNAs and yield a similar stability response. This speculation is because of the lower helix-forming propensity of the natural short sequences as compared to the mutated ones (Figure 5A–D). In Drosophila, mRNA abundance was found to be negatively correlated with transcript size [7]. It is possible that these shorter chloroplast mRNAs are also more abundant and their relatively low stability works in down-regulating their half lives, thereby enabling their pool sizes to stoichiometrically match with those of the lesser abundant but more stable longer transcripts in the same functional complexes. Another interesting component of this result is the logarithmic relationship of average helix-forming propensity of mRNAs with overall mRNA stabilities as well as mRNA lengths (see Figure 5, Additional Files 1 and 2). This means that for mRNAs with lengths beyond a certain limit, the excess length does not contribute towards increasing the helical component of the mRNA, reflecting the need for a certain critical portion of the sequence in loops such that a dynamic structural state can be established vis-à-vis the function. This result has to be considered with caution, because secondary structure predictions are less accurate for long sequences. However, a similar saturating relationship between the integral stability of the mRNA with the helix-forming propensity of that mRNA suggests that this is less likely to be an artifact due to inaccuracies in folding prediction. One could reason that the pattern we observe is species-specific and that other plant genomes might follow different trends, but then the probability of observing a similar response in two phylogenetically distant species is very low. Saturation levels of helix-forming propensity are consistently around 60–65% in both species. Based on this, we surmise that the logarithmic trend is likely to be general.

Additionally, the thermodynamics of secondary structure folding are such that helix-formation decreases enthalpy of the system and is therefore, favored. This is seen in the form of increasing helix-forming propensity with transcript length. Helix-formation, however, also decreases the entropy of the intermediate looped out regions, which becomes the limiting factor at certain mRNA lengths. Further, the kinetics driven by the folding thermodynamics would less favor base-pairing between nucleotides distantly placed from one another on the linear sequence. Pairings between such distantly placed nucleotides might occur but would be unstable and therefore, will not hold. This explains the saturation limit on helix-forming propensity in long mRNAs. Indeed, studies have shown that conformational order present in evolved structures need not arise due to evolutionary optimization, but simply due to intrinsic folding rules of RNA polymers [58]. This is reiterated by the overall similarity between logarithmic trends in helix-forming propensities of mutated and natural mRNAs versus their lengths, despite differences in the details of the trends.

### Protection from Mutations

The simplest explanation for the positive correlation between secondary structure and evolutionary conservation of a site is that being part of helices protects those nucleotide regions from mutations. This mechanism is most critical in the functionally important parts of the protein. This protection may occur both in mRNAs and, as indicated by results, single-stranded DNA during replication. There exists the possibility of a feedback loop: sites that are conserved due to functional constraints will tend to have more secondary structure. This in turn will lead to fewer mutations and therefore, greater conservation. This would result from stabilizing selection for optimal secondary structure(s) because detrimental mutations (resulting in sub-optimal structures) are selected against. In addition, some sites should also be more constrained than others because they are complementary to functionally conserved sites. Hence, some highly conserved sites might be so, not because of their functional importance in the protein, but as part of packing-protection constraints in the mRNA or DNA during single stranded periods. This probable effect at the level of DNA suggests that substitutions are non-randomly distributed with respect to their functional impacts. Hence, the protecting mechanisms that we describe here bias the distribution of mutations towards favorable ones. If correct, this mechanism results in a pattern of adaptive evolution where the secondary structure of nucleic acid sequences, prior to natural selection, filters mutations, a pattern that follows neo-Lamarckian principles because the spectrum of potential mutations is *a priori *biased. This likelihood of this mechanism especially increases while viewing our observation in light of the fact that spontaneous chemical changes occur at a greater rate on single- than double-stranded RNA, quite analogous to mutations on DNA [10]. This means that secondary structure simply by virtue of causing certain regions to be duplex versus certain others, results in low and high mutation regimes. This scenario, where secondary structure also involves evolutionary conservation, is therefore, a relatively adaptive one, as compared to one where this association was absent, where detrimental mutations are not only minimized but also certain beneficial mutations are allowed. Thereby, a mechanism evolves where structure biases the mutational distribution in a way that the positive effects of mutations are more likely. We term this as 'Lamarckian'. It is interesting to note that this pattern could arise, as noted above, from stabilizing selection for an optimal secondary structure, which secondarily would result in a functionally biased mutation spectrum for RNA or DNA coding for proteins.

### Molecular aspects of life history strategies

We found three types of mRNA species in respect to associations between site conservation and formation of secondary structure: those protecting conserved sites from mutations, a smaller group exposing them, and a group where presumably the pressures leading to the former two types are balanced, resulting in more than expected statistically non-significant cases. This suggests that in all mRNAs, and especially in the latter group, both types of strategies exist at different sites in the same mRNA. These might associate with amino acids and protein domains, as well as their function. More detailed analyses at this level could reveal the adaptive strategies for different types of sites and proteins, whether adaptive evolution occurs there by drastic or gradual changes. It is possible that protection of conserved sites promotes gradual adaptive evolution. Their exposure probably causes mutations that are very detrimental, majority of which are filtered out by natural selection and the remaining few which are likely to affect function, might promote saltatory evolution. Since ample sequence data are available, future explorative analyses focused on this issue will surely lead to deeper insights into mRNA and protein functions, and their adaptive evolutionary strategies.

While at the level of mRNA species, we do not yet understand the rationale behind the different types of correlations observed between site variability and stemminess, our preliminary analyses reveal some clues at the level of plant species. However, note that in principle, analyses considering phylogenetic relatedness and larger species numbers are required for assessing the phenomena we preliminarily describe here below. We find that for 66% of mRNAs, '*r*-strategist' species protect the conserved sites more than '*K' *species. This tendency is significant at P < 0.05 (2 tailed t tests) for one mRNA, psbL, but reversed in psbK. These results indicate that minimization of mutations are higher in relatively smaller plants (*r*-strategists), following the general principles of minimization of metabolic costs [8]. In the same plants, this tendency is particularly strong for large mRNAs, which suggests that cost minimization is enhanced for synthesis of costly (large) proteins. The opposite is true for plants which are relatively more *K*-strategists. If one considers that exposing functionally conserved sites to mutations is a strategy that favors saltatory evolution, and that small proteins have more regulatory and large ones more catalytic, basic maintenance functions, then this suggests that *r*-strategists maximize evolutionary potential for regulatory functions, but are relatively conservative at the level of house keeping functions. We find that the opposite is true for *K*-strategists. These results are in line with the view that the large numbers of offspring in *r *strategists enable many unsuccessful evolutionary "experiments" to be done at the level of regulatory functions, while this approach is not tenable for large plants with low offspring numbers.

Experimenting with large proteins is however more cost effective in *K*-strategists with large body sizes, because they have the reserves that enable them to cope with such costs, including the death of body parts, without killing the individual. The results on associations with domestication level, which suggest that the molecular evolutionary strategy of naturally *K*-selected plants becomes more *r*-like by domestication, presumably because domestication increases reproductive output, are more speculative. This is not only because our rankings of domestication levels between different species are difficult to justify in a formal way, but also because these analyses should be done separately on different groups of plants, such as eudicotyledons and others, or plants grown for their seeds versus those grown for other edible body parts. However, as the majority of domesticated plants are selected for increased reproductive output, the convergence between natural *r*-strategists and domestication in *K*-strategists seems plausible. It also strengthens the hypothesis that molecular adaptive evolutionary strategies (protecting or exposing functionally important sites to mutations) associates with relative investments in reproduction.

## Conclusion

1. High sequence variability, particularly at third codon positions, inversely correlates with length adjusted messenger RNA stability, highlighting the functional and non-neutral aspects of synonymous site variation. This is also in line with synonymous codon usage bias which favors AT-richness at third codon positions.

2. *In silico *mutations of mRNAs results in destabilization of longer mRNAs but not of shorter mRNAs, which could explain apparently contradictory results in change in folding stabilities upon randomization of RNAs.

3. Our analyses also reveal transcript length as an important factor, which coupled with evolutionary variability controls secondary structure stability. This insight could substantially contribute to RNA sequence and structure optimization studies that did not consider the effects of RNA length.

4. Some highly conserved sites might be so not because of their functional importance in the protein, but as part of packing-protection constraints at the nucleic acid level.

5. Protection by secondary structure from mutations results in a pattern of adaptive evolution where the secondary structure of nucleic acid sequences prevents detrimental mutations, prior to or in addition to stabilizing selection on optimal secondary structures, a pattern that follows neo-Lamarckian principles.

6. In specific mRNAs for some plants, functionally conserved sites are exposed to smutations, which might lead to saltatory adaptive evolution.

7. Protection from mutations at conserved sites is more widespread in *r*-strategists than *K*-strategists, perhaps because of greater mutation minimization pressures in smaller organisms.

8. The above point is enhanced in *r*-strategists for large proteins, and for smaller proteins in *K*-strategists. This might reflect adaptive strategies, the first leading to saltatory evolution in *r*-strategists, the other to gradual adaptive specialization in *K*-strategists.

9. Among *K*-strategists, domestication causes them to resemble *r*-strategists in terms of molecular adaptive strategies.

## Methods

### Sequence Dataset

RNA-synthesis (sense) strand sequences were extracted from NCBI [59] using in-house PERL scripts for thirty-five genes (atpB, petA, petL, rps11, rpl14, rpl16, rps3, rps19, atpI, atpH, rpoB, rps2, rps7, psbK, psbL, psbE, psbF, rps18, rpl20, psbH, psbN, psbB, psaA, psaB, psaC, atpE, psbA, psbD, psaJ, clp, atpF, psbC, rpl32, psbI, and rps8) of 17 plant species (*Oryza sativa *(*japonica *cultivargroup) (NC_001320; [60]); *Triticum aestivum *(NC_002762; [61]); *Zea mays *(NC_001666; [62]); *Calycanthus floridus var. glaucus *(NC_004993; [63]); *Arabidopsis thaliana *(NC_000932; [64]); *Pinus koraiensis *(NC_004677; [65]); *Pinus thunbergii *(NC_001631; [66]); *Nephroselmis olivacea *(NC_000927; [67]); *Marchantia polymorpha *(NC_001319; [68]); *Mesostigma viride *(NC_002186; [69]); *Chlorella vulgaris *(NC_001865; [70]); *Chlamydomonas reinhardtii *(NC_005353; [71]); *Psilotum nudum *(NC_003386; [72]); *Chaetosphaeridium globosum *(NC_004115; [73]); *Physcomitrella patens subsp. patens *(NC_005087; [74]); *Spinacea oleracea *(NC_002202; [75]) and *Adiantum capillus-veneris *(NC_004766; [76]) belonging to the family *Viridiplantae*.

**Figure 6:**
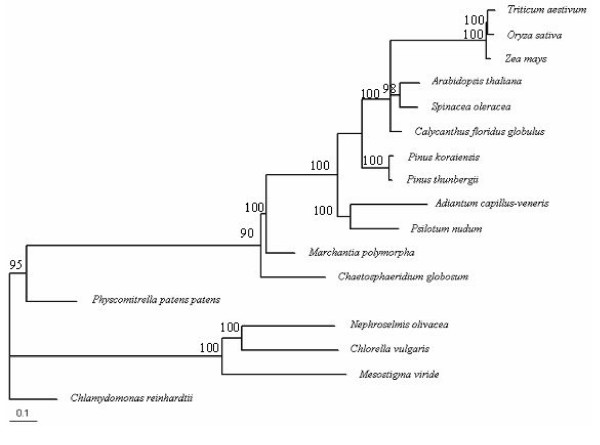
**Chloroplast phylogeny of seventeen plant species**. Phylogeny was calculated by using nucleotide sequence alignments of 35 genes from dataset IV. The branch lengths indicate the relative number of substitutions per site. Non-embryophytic land plants (*Mesostigma viride*, *Chaetosphaeridium globosum*, *Nephroselmis olivacea*, *Chlamydomonas reinhardtii*, and *Chlorella vulgaris*) were defined as out groups and phylogeny was rooted using them. Bootstrap values indicating branch support are marked next to the branches. Bootstrapping was done by generating 100 replicates of the dataset using "seqboot" program in PHYLIP v.3.67. Phylogenies were generated from these replicates again using the "dnaml" criterion and then drawing a consensus using "consense" option.

We considered as the anti-sense (coding) strand, the strand that coded for a majority of 23 out of 35 sampled genes. For the 12 sense-stranded coded genes (clpP, psbE, psbF, petL, petA, psaJ, psbC, psbB, psbD, psbK, psbH, rps18), we considered the sequence as it is and for the remaining genes (atpI, atpF, atpH, atpB, atpE, psaA, psaB, psaC, psbA, psbL, psbN, psbI, rpoB, rpl14, rpl16, rpl20, rpl32, rps11, rps19, rps2, rps3, rps7, rps8), we analyzed the reverse complementary sequence. This combination of 17 species and 35 genes was chosen because it consists of an ample number of species and at the same time, a sufficiently large number of homologous genes (concatenated alignment of 27,465 nucleotides) for building the chloroplast phylogeny. We used ClustalW [77] for individually aligning the protein-coding genes, and further, concatenated them to create the necessary dataset for building a phylogeny. Our homology definition criteria included both paralogous as well as orthologous genes, and were judged purely on the basis of NCBI annotations of genes. The phylogeny we used here is shown in Figure 6 along with bootstrap values.

### mRNA secondary structure and stability

The sense strand (RNA-like) sequences of all thirty-five genes were folded as RNA as well as DNA using RNA and DNA folding programs, respectively, from mFold server [54,78]. We recorded the negative of the free energy (-ΔG; delta-G in kcal/mol) of the folded structures, which we will refer to as integral stability. All alternative structures within 50% sub-optimality of the most stable structure were considered and the average ΔG over all these structures was computed.

We assumed that doing so would yield a fairly realistic ΔG, minimize the effects of inaccurate predictions by mFold and reduce sampling bias. This average estimate was further averaged across all the seventeen species, for each mRNA. The secondary structure stability of the mRNA is highly correlated with mRNA length (r = 0.99, see Additional File 2). Therefore, we calculated residual ΔG by performing linear regression between mRNA length as the independent variable and average (-ΔG) as dependent variable. We deducted the value predicted by this linear relationship of -ΔG with mRNA length from the observed average (-ΔG). This residual ΔG quantifies the intrinsic stability due to the information contained in the mRNA or DNA sequence, independent of the length of the sequence. It is therefore, comparable across mRNAs of different lengths. This is the estimate of stability which we use for all our analyses and we will refer to it as length-adjusted residual stability. The evolutionary significance of residual over integral stability is further clarified in the Discussion section.

### Site-specific evolutionary variability measures for genes

Phylogeny was inferred using "dnaml" program in PHYLIP v 3.8.1 [79] on the concatenated nucleotide sequence alignment of thirty-five genes across 17 species. The bootstrapped phylogeny used to infer site-specific evolutionary variability levels is shown in Figure 6). Four average substitution rate categories were chosen: M1 (λ = 0.1); M2 (λ = 0.2); M3 (λ = 0.3) and M4 (λ = 0.4) based on prior probabilistic indications of site-specific average rates by Hidden Markov Models while inferring the phylogeny. We obtained individually for each gene, the relative most likely fits to these four rate categories for all the 35 genes using the maximum likelihood approach of "dnaml" program. The read-out we got from this tool was for example, as follows: 30% of the sites in a gene fits rate category M1 while 50% of the gene fits category M2.

We also compared these substitution rate category fits to read-outs obtained using another model available within a Bayesian Monte Carlo Markov chain framework [80,81]. This approach accounts for scenarios where sites can evolve in qualitatively different ways, i.e. follow pattern heterogeneity in addition to evolving at different rates. We found that the fits of sites to these substitution patterns correlated significantly in the expected direction with %fits of sites to the above mentioned rate categories from PHYLIP (see Additional File 5)

We used Pearson's correlation coefficients to estimate the strength of the correlation between site variability and secondary structure (termed "stemminess") at the site, for each gene in each species. We also used the more robust and conservative Spearman's non-parametric rank correlation test, in addition to the parametric test, to estimate correlations between sequence variability and folding stability.

### Simulating evolution of mRNA sequences

In order to study the effect of sequence variability on mRNA stability, specifically, in terms of helix-forming propensity, we introduced mutations in the mRNAs of a randomly chosen species, *Psilotum nudum *and of another species, phylogenetically distant from it, *Nephroselmis olivacea*. We chose to calculate the helix-forming propensity in our primary measure of stability (ΔG) in order to explore how mutations affect sites with different levels of evolutionary variability in terms of their propensities to form helices or to be part of loops. Mutations were introduced *in silico *by changing the nucleotide at each site with a probability proportional to a substitution rate category that it best fits to (M1, M2, M3 or M4), obtained from PHYLIP v 3.8, along with a transition:transversion bias of 2:1. Fifty such mutation rounds were performed for each mRNA to yield fifty mutated sequences. Helix-forming propensities were averaged across all the fifty mutants to generate a single value representing the mutated mRNA helix-forming propensity. This process mutated on average 17% of the nucleotides in each sequence.

We compared the helix-forming propensities of natural mRNAs with those of the mutants, vis-à-vis mRNA length, for all sites as well as separately for each variability category of sites for the two species, *Psilotum nudum *and *Nephroselmis olivacea*. For these analyses, we sampled nine mRNAs of different lengths: psbF (120 bases), psbK (177 bases), rps18 (227 bases), rps9 (278 bases), rpl14 (368 bases), rps3 (641 bases), petA (965 bases), psbB (1526 bases) and psaA (2244 bases) and studied the change in helix-forming propensity upon mutations across different mRNA lengths. We also include in our comparisons, helix-forming propensities of randomized mRNAs of the same length.

Fifty mononucleotide randomizations were performed for each of the nine mRNAs and their helix-forming propensities were averaged over these fifty simulated sequences (see [54] for a justification of single base-shuffling randomization approach)

### Life history strategies and domestication

We defined all species belonging to Spermatophyta as relatively fitting the *K*-strategists life history strategy, due to their larger sizes, longevities, and lower offspring numbers.

This group also includes the large fern species of our sample. Other non-Spermatophyte plants, which were of relatively smaller sizes and longevities, and larger offspring numbers, were considered from an ecological point of view as relatively *r*-strategists (see for review of the concepts [82]). Body size, as a factor for classifying *K*- and *r*-strategists among plant species, was also used by Barradas et al. [83]. The usage of longevity and offspring numbers as classification factors for life-history strategies in plants, is adapted from the microbial framework provided by Andrews and Harris [84]. For analyses exploring associations with levels of domestication, we only considered Spermatophyta. Species were crudely ranked from no domestication to high domestication in an increasing order, according to the level of alteration in the plant as compared to ancestral wild plants (i.e. maize got the highest level of domestication, and wild pines the lowest. *Triticum aestivum *got a relatively high level due to its hybrid origins).

## Authors' contributions

NMK conceived the idea of exploring chloroplast gene sequence-structure relationship, carried out correlation and simulation analyses and drafted the manuscript. BJR added the idea to perform simulations which would reveal exactly how mutations affect secondary structure. HS contributed the idea of exploring variation in molecular strategies with respect to ecological life-styles. NMK, HS and BJR have read and given final approval of the version to be published.

## Supplementary Material

Additional File 1 Correlation between stem-forming propensities of mRNAs vs. length of the mRNA for natural, random and mutated sequences of *Nephroselmis olivacea *Nine mRNAs of varying lengths (psbF (120 bases), psbK (177 bases), rps18 (227 bases), rps9 (278 bases), rpl14 (368 bases), rps3 (641 bases), pet A (965 bases), psbB (1526 bases) and psaA (2244 bases)) belonging to the species *Nephroselmis olivacea *(NC_000927) are analyzed here. For mutated mRNAs, each point represents average stem-forming propensity over fifty such mutated sequences and over at least 50% sub-optimal alternative structures for each mutated sequence. The random mRNA stability is an average over stabilities of fifty mononucleotide randomizations while maintaining the sequence length. Click here for file

Additional File 2 Relationship between A. Integral Stability (-ΔG) and mRNA size and B. Average stem-forming propensity (%) and integral stability. In A., the y-axis is the length of the mRNA and x-axis is the negative of the folding stability (ΔG) in kcal/mol. The dataset consists of seventeen species and thirty-five genes (see Methods). For each mRNA, alternative structures that were at least half as stable as the most stable structure were considered and stabilities were averaged across all these structures. Further, the ΔGs were averaged across all the seventeen species. Similarly, the relative propensity of mRNA regions to form helices were averaged over the top 50% sub-optimal alternative structures and further across all the seventeen species (y-axis in B.) Click here for file

Additional File 3 t statistics of the regression slopes between stem formation at sites and variability level of the site, for each mRNA in each plant species. The second row indicates the strand on which the genes are encoded: AS (anti-sense) or S (sense). The third last row indicates the number of plant species for which this association is negative in that mRNA. The row before last indicates the correlation between the negative of ΔG of the mRNA (as a proxy of gene length) and the t statistics in the column above. The last row indicates the Spearman's rank correlation coefficients for association between the t-statistics for correlations of stem-formation with variability level of each mRNA with the domestication level of species. The fourth last column indicates the number of negative t statistics in that plant; the third last column indicates the correlation between the ΔG of the mRNA and the t statistics in that plant species, the second last column indicates the presumed life strategy of that plant species (*r *or *K*), and the last column indicates the presumed level of domestication in Spermatophyta, estimated based on the level of alterations from the wild plant. The numbers in bold are significant according to two-tailed tests for correlations, and according to sign tests when numbers of negative correlations are counted. Click here for file

Additional File 4 Evolutionary variability effects on integral and residual, length-adjusted stability. Correlations between the absolute numbers of sites fitting the four variability categories: M1, M2, M3, and M4 (Y-axis) and A. integral stability (-ΔG) and B. residual, length-adjusted stability (-ΔG) are shown here. Negative of mRNA stabilities were averaged over the top 50% sub-optimal alternative structures and further across all the seventeen species. Residuals were calculated by treating length as the independent variable and the negative of stability (-ΔG) as the dependent variable (see Methods). Click here for file

Additional File 5 Correlations between site-specific substitution rate categories and patterns calculated by PHYLIP and Bayes Phylogenies tools. Correlations between percent fits of all 35 genes to site-specific rate categories (M1, M2, M3 and M4) estimated under the single-rate heterogeneity model (dnaml) available in PHYLIP and substitution patterns (P0-P3, Figures A-D, respectively) estimated under the pattern-heterogeneity model available in the Bayesian Monte Carlo Markov chain framework for phylogenetic inference [75]. Click here for file
