# Supplementary figures and images for: Relationship between mRNA secondary structure and sequence variability in Chloroplast genes: possible life history implications
Source: BMC Genomics. 2008 Jan 28;9:48. doi: 10.1186/1471-2164-9-48 (PMC2276208; doi:10.1186/1471-2164-9-48)

A B
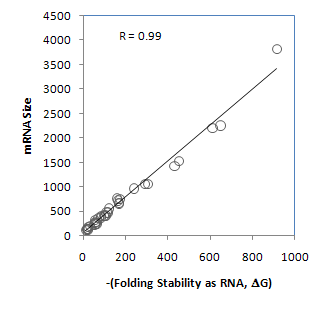

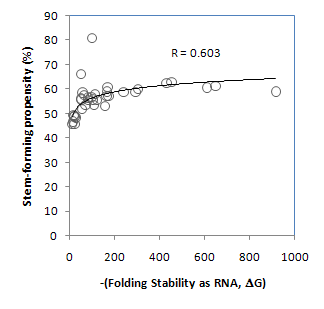

Supplement: Additional File 2 — Relationship between A. Integral Stability (-ΔG) and mRNA size and B. Average stem-forming propensity (%) and integral stability. In A., the y-axis is the length of the mRNA and x-axis is the negative of the folding stability (ΔG) in kcal/mol. The dataset consists of seventeen species and thirty-five genes (see Methods). For each mRNA, alternative structures that were at least half as stable as the most stable structure were considered and stabilities were averaged across all these structures. Further, the ΔGs were averaged across all the seventeen species. Similarly, the relative propensity of mRNA regions to form helices were averaged over the top 50% sub-optimal alternative structures and further across all the seventeen species (y-axis in B.) [file 1471-2164-9-48-S2.doc]

A B


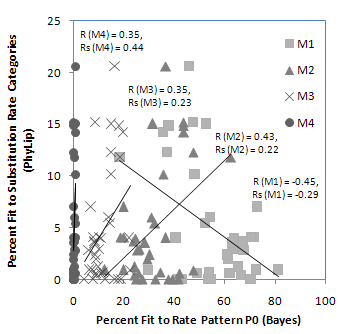

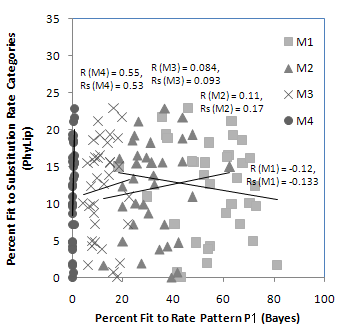


C D
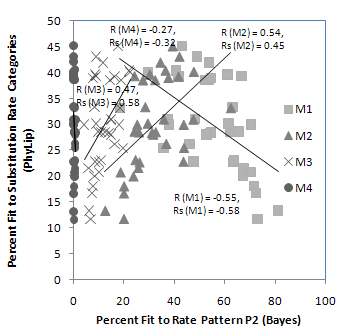

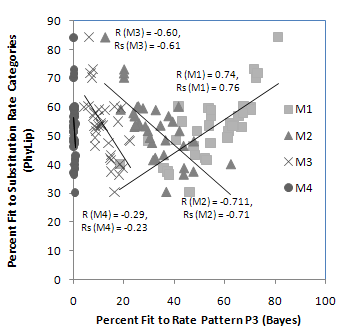

Supplement: Additional File 5 — Correlations between site-specific substitution rate categories and patterns calculated by PHYLIP and Bayes Phylogenies tools. Correlations between percent fits of all 35 genes to site-specific rate categories (M1, M2, M3 and M4) estimated under the single-rate heterogeneity model (dnaml) available in PHYLIP and substitution patterns (P0-P3, Figures A-D, respectively) estimated under the pattern-heterogeneity model available in the Bayesian Monte Carlo Markov chain framework for phylogenetic inference [75]. [file 1471-2164-9-48-S5.doc]
