# Supplementary material for: Relationship between mRNA secondary structure and sequence variability in Chloroplast genes: possible life history implications
Source: BMC Genomics. 2008 Jan 28;9:48. doi: 10.1186/1471-2164-9-48 (PMC2276208; doi:10.1186/1471-2164-9-48)
Supplement: Additional File 3 — t statistics of the regression slopes between stem formation at sites and variability level of the site, for each mRNA in each plant species. The second row indicates the strand on which the genes are encoded: AS (anti-sense) or S (sense). The third last row indicates the number of plant species for which this association is negative in that mRNA. The row before last indicates the correlation between the negative of ΔG of the mRNA (as a proxy of gene length) and the t statistics in the column above. The last row indicates the Spearman's rank correlation coefficients for association between the t-statistics for correlations of stem-formation with variability level of each mRNA with the domestication level of species. The fourth last column indicates the number of negative t statistics in that plant; the third last column indicates the correlation between the ΔG of the mRNA and the t statistics in that plant species, the second last column indicates the presumed life strategy of that plant species (r or K), and the last column indicates the presumed level of domestication in Spermatophyta, estimated based on the level of alterations from the wild plant. The numbers in bold are significant according to two-tailed tests for correlations, and according to sign tests when numbers of negative correlations are counted. [file 1471-2164-9-48-S3.doc]

***Table 1***  t statistics of the regression slopes between stem formation at sites and variability level of the site, for each mRNA in each plant species.

| Species | atpB | atpE | atpF | atpH | atpI | clpP | petA | petL | psaA | psaB | psaC |
| --- | --- | --- | --- | --- | --- | --- | --- | --- | --- | --- | --- |
| Strand | AS | AS | AS | AS | AS | S | S | S | AS | AS | S |
| Adiantum capillus-veneris | 0.3 | -0.1 | -0.5 | -1.5 | -0.1 | -1.4 | 1.24 | -1 | -0.2 | 0.4 | 1.1 |
| Arabidopsis thaliana | 0.1 | 0.6 | -0.2 | -0.2 | 0.1 | -0.7 | 1.32 | 0.6 | 0.07 | -0.1 | 1 |
| Calycanthus | 0.1 | 0.8 | 1.1 | 0.2 | -2.1 | -1.1 | 1.39 | 1.5 | -0.2 | -0.2 | 0.6 |
| Chaetosphaeridium globosum | 0.8 | 0.5 | 0.9 | -1.2 | -1.7 | 1 | 0.54 | -0.3 | -0.9 | -1 | -0.1 |
| Chlamydomonas reinhardtii | 0.4 | **-2.5** | 0.0 | -1.6 | -1.1 | -0.4 | 0.0 | -0.3 | -1.1 | -3.1 | **-2.2** |
| Chlorella vulgaris | 1 | 0.9 | 0.9 | -1.6 | 0.5 | -2 | 1.55 | 0.2 | -3.1 | -2.4 | -0.3 |
| Marchantia polymorpha | -2 | -2 | 0.1 | -1.5 | 0.9 | -1.7 | 0.88 | 0.1 | -2.3 | -1.6 | 1.4 |
| Mesostigma viride | -1.7 | -1.7 | -2.3 | -0.8 | -0.7 | 1.5 | -0.6 | -0.9 | -1.7 | -1.2 | 0.7 |
| Nephroselmis olivacea | 1.6 | 0.9 | 0.2 | -1.3 | -1 | 1.9 | 0.12 | 1.8 | 0.04 | 1.3 | 1.3 |
| Oryza sativa | 0.4 | 0.2 | -1.3 | 0.3 | 0 | -0.1 | 0.56 | 1.5 | 0 | -0.6 | -0.3 |
| Physcomitrella patens | 0.1 | -0.2 | 0.5 | -0.4 | -0.5 | -0.1 | 0.49 | 0.9 | -1 | -0.8 | 1.9 |
| Pinus koraiensis | -1.3 | -2.1 | 1.1 | -0.3 | 0.8 | -1.4 | 0.39 | **2.2** | 0.16 | -1.3 | 1.9 |
| Pinus thunbergii | -1.2 | -1.5 | 0.5 | -1.6 | 0.3 | -0.5 | 1.29 | 2.3 | -0.2 | -1 | 1 |
| Psilotum nudum | -1.5 | -1.7 | 0.4 | -1.7 | 0.2 | -0.9 | 0.21 | -0.4 | -0.6 | -1.6 | 0.1 |
| Spinacia oleracea | 1 | 1.2 | -0.7 | 0 | 0.3 | -0.4 | -1.5 | 0.8 | -0.3 | -0.2 | 1.4 |
| Triticum aestivum | 0.5 | 0.7 | -0.4 | -0.3 | 0 | -1.5 | 1.65 | 1.3 | -0.3 | -1 | 1 |
| Zea mays | 0.6 | 0.2 | 0.4 | 0 | -0.1 | -1.1 | 1.5 | -0.3 | -0.8 | -0.6 | -0.2 |
| Negative | 5 | 8 | 6 | 13 | 10 | 14 | 2 | 6 | 14 | 15 | 5 |
| rΔG | 0.15 | 0.29 | 0.11 | 0.06 | 0.38 | -0 | 0.30 | **0.49** | 0.77 | 0.74 | 0.23 |
| rs Domestication | 0.9 | 0.4 | -0.7 | 0.4 | -0.6 | 0 | 0.48 | -0.8 | -0.7 | 0.2 | -0.6 |

| Species | psaJ | psbA | psbB | psbC | psbD | psbE | psbF | psbH | psbI | psbK | psbL |
| --- | --- | --- | --- | --- | --- | --- | --- | --- | --- | --- | --- |
| Strand | S | AS | S | S | S | S | S | S | AS | S | AS |
| Adiantum capillus-veneris | -0.4 | -0.6 | -0.4 | -0.3 | -0.3 | 0.6 | 0.47 | 0.3 | 0.7 | -0.5 | -2 |
| Arabidopsis thaliana | 0.4 | -0.8 | -1.3 | -0.4 | -0.5 | -0.5 | 0.11 | -0.1 | -0.1 | -2.3 | 0.2 |
| Calycanthus floridus glaucus | -0.9 | -1.5 | 0.4 | -2.2 | -0.7 | 0.3 | 1.33 | 1 | 0.7 | -0.8 | 0.4 |
| Chaetosphaeridium globosum | 0.4 | -2 | -1.1 | -0.1 | 0.8 | -0.2 | -0.2 | 0.1 | 1.2 | 1.2 | 0 |
| Chlamydomonas reinhardtii | -1.2 | **-2.5** | **-2.1** | -0.8 | -1.1 | -1.8 | -1.2 | -0.5 | 0.9 | -0.3 | 0.4 |
| Chlorella vulgaris | 0 | -0.8 | -0.7 | -1.4 | -1.8 | -0.1 | -0.6 | -1.2 | -0.6 | 1 | -0.1 |
| Marchantia polymorpha | -0.6 | -1.8 | -0.7 | -0.1 | -1.4 | 0.6 | -0.5 | 0.7 | 0.8 | 1.2 | -0.7 |
| Mesostigma viride | 0.8 | -2.7 | -1.2 | -2.3 | -1.2 | -1.6 | -1.3 | -1.5 | 2.2 | 0.2 | -0.7 |
| Nephroselmis olivacea | -0.1 | -1.9 | -0.8 | -0.8 | 0 | 0.1 | -1.6 | -0.4 | 1.3 | -0.9 | -0.1 |
| Oryza sativa | -1.3 | -2.7 | -1.8 | -2 | -0.6 | 0.6 | -0.2 | 0.9 | 0.7 | -1.4 | 0.4 |
| Physcomitrella patens | -0.8 | -2.1 | -0.9 | -1.4 | -1.4 | -1.3 | 0.39 | -0.4 | 0.7 | 0.6 | -0.3 |
| Pinus koraiensis | 1.4 | -0.4 | -1 | -0.4 | -1.6 | -0.7 | 0.03 | 0.4 | 1.4 | -0.4 | 0.1 |
| Pinus thunbergii | -0.5 | -0.6 | -0.1 | 0.3 | -1.7 | -0.7 | -0.4 | 0.5 | 1.6 | -0.4 | 0.6 |
| Psilotum nudum | -0.3 | -3.5 | 0.5 | -0.5 | -1.5 | 0.1 | -0.2 | -1.1 | 0.1 | **-2** | -0.1 |
| Spinacia oleracea | -0.5 | -1 | 0.7 | -0.2 | -1.5 | 0.8 | 0.6 | 0.3 | -1.2 | 0.1 | -0.2 |
| Triticum aestivum | -0.4 | -2.1 | -1.5 | -2.2 | -0.7 | 0.2 | -0.5 | -0.1 | -1 | -1.7 | 0.4 |
| Zea mays | -1.4 | -3.5 | -1.5 | -2.2 | 0 | 0.5 | -0.7 | 0.6 | -1.7 | -2.8 | 0.4 |
| Negative | 12 | 17 | 14 | 16 | 14 | 8 | 11 | 8 | 5 | 11 | 9 |
| rΔG | -0.3 | 0.29 | 0.21 | -0.02 | 0.38 | 0.3 | 0.01 | 0.45 | 0.04 | 0.28 | 0.15 |
| rs Domestication | -0.5 | -0.9 | -0.6 | -0.7 | 0.68 | 0.66 | -0.5 | -0.1 | -0.8 | -0.6 | 0.1 |

| Species | psbN | RNApolB | rpl14 | rpl16 | rpl20 | rpl32 | rps11 | rps18 | rps19 | rps2 | rps3 |
| --- | --- | --- | --- | --- | --- | --- | --- | --- | --- | --- | --- |
| Strand | AS | AS | AS | AS | AS | AS | AS | S | AS | AS | AS |
| *Adiantum capillus-veneris* | 0.6 | **6** | 1.1 | 0.4 | 1 | -1.1 | -0.2 | 0.8 | -0.3 | -1.1 | 0.6 |
| *Arabidopsis thaliana* | 0.3 | 0.9 | 0.2 | -1.1 | 0.9 | -1 | -0.1 | 1 | -1 | -1.1 | 0.3 |
| *Calycanthus floridus glaucus* | -0.1 | **4.5** | -0.1 | 0 | **2.1** | -0.1 | 0.64 | 1.2 | 0.59 | -0.9 | 0.7 |
| *Chaetosphaeridium globosum* | 0.5 | 1 | **2.4** | 0.2 | -1.3 | 1 | -1.5 | 1.9 | -0.1 | -1.5 | -1.2 |
| *Chlamydomonas reinhardtii* | -1.8 | -0.5 | 0.4 | -1.8 | -0.4 | -0.8 | -0.7 | 0.1 | 0.3 | -1.2 | -0.4 |
| *Chlorella vulgaris* | -1 | 0.6 | 0.7 | -0.3 | -1.1 | 0.8 | 0.25 | 0.5 | -0.2 | -0.3 | -0.8 |
| *Marchantia polymorpha* | -1 | 1.9 | 0.3 | -0.6 | 0.7 | -0.5 | -0.5 | 0.8 | -1.1 | **-3.8** | -0.4 |
| *Mesostigma viride* | 1.3 | 1.9 | 0.2 | -0.8 | -0.3 | 1.3 | -0.9 | -0.7 | -0.9 | -1 | 0.9 |
| *Nephroselmis olivacea* | -0.8 | 1.2 | 0.7 | 0.7 | 0.4 | **-2** | 0.85 | 1 | 1.69 | -0.1 | -0.6 |
| *Oryza sativa* | 0.5 | **2.5** | 0.9 | -0.4 | -0.7 | 0.4 | 1.89 | -0.1 | -1.2 | 0.2 | -0.1 |
| *Physcomitrella patens* | -0.6 | **3.9** | -0.6 | 0.7 | -1.9 | -1.4 | -0.1 | 1.5 | -1.5 | **-2.4** | 0 |
| *Pinus koraiensis* | -0.2 | 0.9 | 1.5 | -0.7 | 0.8 | 1.3 | -0.2 | 0.9 | -0.9 | -1.4 | -1 |
| *Pinus thunbergii* | 0 | -0.1 | 1.6 | -0.6 | **2.4** | 0.6 | **2.14** | 0.8 | -0.4 | -0.9 | -0.4 |
| *Psilotum nudum* | -0.7 | **3.2** | -0.3 | -0.5 | -0.2 | -1.9 | -1.2 | 1 | -0.7 | -1.9 | **2.4** |
| *Spinacia oleracea* | -0.2 | **7.2** | 0.9 | -0.4 | 1.4 | 1.1 | 1.13 | 1.5 | -1.6 | -0.1 | 0.3 |
| *Triticum aestivum* | 0.2 | 1.8 | 1.7 | -1.1 | -0.8 | 0.2 | 0.9 | 1.2 | -1.2 | **-2** | -0.2 |
| *Zea mays* | 0.1 | **2.6** | 0.4 | -0.1 | -0.6 | -1 | -0.5 | 1.2 | -1.8 | **-3** | 0.9 |
| Negative | 10 | **2** | **3** | 12 | 9 | 10 | 10 | **2** | **14** | **16** | 9 |
| rΔG | 0.08 | 0.02 | 0.27 | 0.07 | **0.6** | -0.14 | **0.6** | 0.01 | **0.5** | 0.42 | -0.2 |
| rs Domestication | 0.46 | 0.52 | 0 | 0.15 | **-0.7** | -0.5 | -0.2 | 0.4 | -0.8 | -0.3 | 0.5 |

| Species | rps7 | rps8 | Neg. | r DG | Life history | Domestication |
| --- | --- | --- | --- | --- | --- | --- |
| Strand | AS | AS |  |  |  |  |
| *Adiantum capillus-veneris* | -1.2 | **-3.4** | 20 | **0.4** | K |  |
| *Arabidopsis thaliana* | 0.8 | -1.3 | 18 | 0.1 | K | 3 |
| *Calycanthus floridus glaucus* | 1.1 | -0.4 | 14 | 0.2 | K | 2 |
| *Chaetosphaeridium globosum* | -1.5 | -0.9 | 19 | -0.2 | r |  |
| *Chlamydomonas reinhardtii* | 0.1 | -0.4 | **26** | -0.3 | r |  |
| *Chlorella vulgaris* | -0.4 | -1.6 | **22** | -0.2 | r |  |
| *Marchantia polymorpha* | 0 | -1.9 | 21 | 0 | r |  |
| *Mesostigma viride* | -0.2 | -1.4 | **25** | -0.1 | r |  |
| *Nephroselmis olivacea* | -0.8 | -0.4 | 16 | 0.1 | r |  |
| *Oryza sativa* | -0.8 | -0.6 | 20 | 0.1 | K | 5 |
| *Physcomitrella patens* | 0.2 | -1.5 | **22** | 0.1 | r |  |
| *Pinus koraiensis* | 1.1 | -0.5 | 18 | -0.1 | K | 1 |
| *Pinus thunbergii* | 0.8 | -0.5 | 20 | -0.2 | K | 1 |
| *Psilotum nudum* | 1.7 | -1.4 | **24** | 0.2 | K |  |
| *Spinacia oleracea* | 0.7 | -0.9 | 16 | **0.4** | K | 4 |
| *Triticum aestivum* | -0.8 | -0.5 | **22** | 0 | K | 6 |
| *Zea mays* | -1.1 | 0.4 | 21 | 0.2 | K | 7 |
| Negative | 9 | **16** |  |  |  |  |
| rΔG | 0.31 | 0.25 |  |  |  |  |
| rs Domestication | -0.9 | 0.2 |  |  |  |  |

The second row indicates the strand on which the genes are encoded: AS (anti-sense) or S (sense). The third last row indicates the number of plant species for which this association is negative in that mRNA. The row before last indicates the correlation between the negative of ΔG of the mRNA (as a proxy of gene length) and the t statistics in the column above. The last row indicates the Spearman’s rank correlation coefficients for association between the t-statistics for correlations of stem-formation with variability level of each mRNA with the domestication level of species. The fourth last column indicates the number of negative t statistics in that plant; the third last column indicates the correlation between the ΔG of the mRNA and the t statistics in that plant species, the second last column indicates the presumed life strategy of that plant species (*r* or *K*), and the last column indicates the presumed level of domestication in Spermatophyta, estimated based on the level of alterations from the wild plant. The numbers in bold are significant according to two-tailed tests for correlations, and according to sign tests when numbers of negative correlations are counted.
