# Supplementary material for: Relationship between mRNA secondary structure and sequence variability in Chloroplast genes: possible life history implications
Source: BMC Genomics. 2008 Jan 28;9:48. doi: 10.1186/1471-2164-9-48 (PMC2276208; doi:10.1186/1471-2164-9-48)
Supplement: Supplementary file 3 [file 1471-2164-9-48-S1.doc]

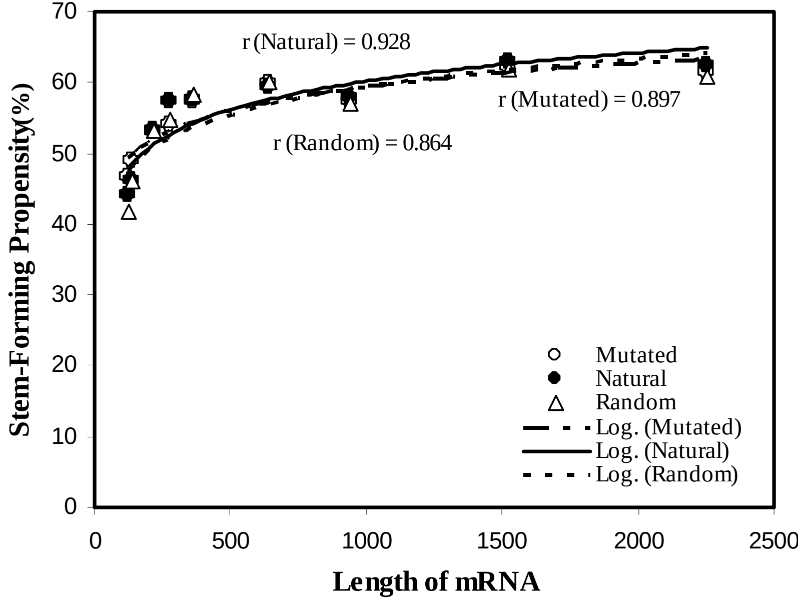

r (Natural) = 0.928
r (Mutated) = 0.897
r (Random) = 0.864
Mutated
Natural
Random
Log. (Mutated)
Log. (Natural)
Log. (Random)
Stem-Forming Propensity(%)
Length of mRNA
0
10
20
30
40
50
60
70
0
500
1000
1500
2000
2500
