# Supplementary material for: Relationship between mRNA secondary structure and sequence variability in Chloroplast genes: possible life history implications
Source: BMC Genomics. 2008 Jan 28;9:48. doi: 10.1186/1471-2164-9-48 (PMC2276208; doi:10.1186/1471-2164-9-48)
Supplement: Additional File 4 — Evolutionary variability effects on integral and residual, length-adjusted stability. Correlations between the absolute numbers of sites fitting the four variability categories: M1, M2, M3, and M4 (Y-axis) and A. integral stability (-ΔG) and B. residual, length-adjusted stability (-ΔG) are shown here. Negative of mRNA stabilities were averaged over the top 50% sub-optimal alternative structures and further across all the seventeen species. Residuals were calculated by treating length as the independent variable and the negative of stability (-ΔG) as the dependent variable (see Methods). [file 1471-2164-9-48-S4.doc]

A	B
